# Cross-cultural adaptation of a Spanish version of a previously validated HPV survey that evaluates dental students’ knowledge, perception and clinical practices in Latin America

**DOI:** 10.1186/s12903-022-02108-2

**Published:** 2022-03-14

**Authors:** Lilliam M. Pinzon, Alan Velazquez, Holdunn Rutkoski, Djin L. Tay, Lara Martel, Carmen Drury, Shauna Ayres, Barbara Dixon, James R. Winkler, Deanna Kepka

**Affiliations:** 1 College of Nursing, Huntsman Cancer Institute, University of Utah, 2000 Circle of Hope, Salt Lake City, UT 84112 USA; 2 Universidad Autonoma de Baja California, Tijuana, Mexico; 3 Caring Health Center, Springfield, MA USA; 4 Utah Department of Health, Salt Lake City, UT USA; 5 Huntsman Cancer Institute, Salt Lake City, UT USA

**Keywords:** Oropharyngeal cancer, HPV vaccination, Latin America, Dental students, HPV survey

## Abstract

**Background:**

The global incidence of oropharyngeal cancer (OPC) is increasing. Dental professionals play a key role in the detection of oral lesions that could lead to cancer. However, scientific-based HPV-OPC visual inspection guidelines are underdeveloped and HPV knowledge and awareness has been reported to be low among dental students and professionals. The present study adapted and performed pretesting of a multi-scale survey evaluating knowledge, perceptions, and clinical practices regarding HPV and HPV-OPC for Latin American Spanish-speaking populations.

**Methods:**

A previously developed questionnaire for English-speaking dental students was translated to Spanish. The questionnaire was administered to first year dental students at two Latin American universities with dental programs. Internal consistencies were measured using Cronbach Alpha. Analyses were conducted in SAS Version 9.4.

**Results:**

Data from a total of 114 students, a majority of the which were female (61%), and Hispanic/Latino(a)/Spanish (91%). The HPV, HPV-OPC, and HPV vaccine knowledge subscales demonstrated good internal consistency, the Cronbach’s alpha was 0.83, 0.75, and 0.86 respectively. The Barriers subscale had a Cronbach’s alpha of 0.93, showing excellent internal consistency. The Clinical Procedures subscale, focused on factors surrounding dental students’ hypothetical clinical practice procedures, had a Cronbach’s alpha of 0.86. The Scope of Practice scale had a Cronbach’s alpha of 0.93.

**Conclusions:**

Ultimately, this survey demonstrated reliability and applicability for the assessment of dental students’ knowledge, perceptions, and clinical practices regarding HPV and HPV-OPC in Latin America.

**Supplementary Information:**

The online version contains supplementary material available at 10.1186/s12903-022-02108-2.

## Background

The global incidence of oropharyngeal cancer (OPC) is increasing, especially among those younger than 45 years [1]. An estimated 9,180 new cases of OPC were reported in Latin America and the Caribbean in 2018 with 80% of these cases occurring in men [2]. The countries with the most OPC cases were Brazil, Venezuela, Cuba, Colombia, Mexico, and Argentina [3]. Epidemiological and in vitro research reports suggest a strong association of human papillomavirus (HPV) with OPC [4], which is further supported by reports from Mexico and Colombia stating that HPV is the attributable factor in 36% of OPC cases [5].

Dental professionals play a key role in the detection of any oral and OPC lesions that could lead to cancer [6]; however, scientific-based HPV-OPC visual inspection guidelines are still underdeveloped. Previous studies suggest that oropharyngeal cancer screening via oral rinse may be a useful tool to detect HPV associated OPC [7, 8]. However, the U.S. Food and Drug Administration (FDA) has yet to approve any tests to detect HPV in the oral cavity [9], making widespread HPV vaccination the leading prevention strategy to reduce the HPV-OPC burden. In an effort to increase HPV vaccination coverage, the FDA has recently approved HPV vaccine Gardasil-9 for men and women aged 27–45 years old [10]. Acknowledging the increase of HPV-OPC incidence, the American Dental Association’s (ADA) recently adopted policy stating that dentists need to be current with HPV-OPC knowledge and support HPV vaccine use and administration [11].

With sufficient knowledge and adequate training on how to educate patients about HPV vaccination, dental providers have the potential to lead HPV-OPC prevention [12]. In fact, administering the HPV vaccine in the dental setting could be a viable option according to a multi-state study of United States (US) dental students, where it was found that a majority of dental and dental hygiene students are open to be trained to administer the HPV vaccine [13]. Thus, it is crucial to evaluate dental professional’s knowledge of, perceptions about, and clinical practices surrounding HPV and HPV vaccination. It is also important to understand what elements of dental school curriculum and clinical practices could improve HPV-OPC education and screening.

Globally, HPV knowledge and awareness has been reported to be low among the dental student, oral health student, and oral health professional populations; however, the construct validity of these instruments is questionable as many were study-specific [14–21]. Likewise, studies that examine HPV knowledge and awareness in Latin American countries are limited, but point to suboptimal levels of knowledge and awareness of HPV in countries such as Brazil and Ecuador [22, 23].

Validated instruments have been developed in the United States for HPV and HPV vaccine communication, advocacy, attitudes, and clinical practices among dental providers [18, 24–26]. Researchers from the University of Minnesota utilized a validated 32-item survey instrument designed to measure head and neck surgeon’s knowledge, attitudes, and practices regarding HPV communication and vaccine advocacy with dentists and dental hygienists, and found a lack of knowledge about HPV and HPV vaccination [18]. However, all of these scales were developed for English-speaking respondents, and to our knowledge, none have been tested for applicability to Latin America, a region with a high burden of emerging HPV-OPC and low HPV knowledge and awareness.

It is crucial that the next generation of Latin American dental professions have the tools needed to improve the health and well-being of their patients through increasing HPV vaccination coverage. This can be achieved through HPV-OPC patient education [26], HPV vaccination recommendation, and preventative OPC screening [27]. Having a validated Spanish instrument evaluating KPCP for HPV and HPV-OPC would help identify areas of where Latin American dental schools could improve curriculum and secondarily help improve HPV-OPC prevention among their populations.

The present study aimed to perform a cross-cultural adaptation of a multi-scale questionnaire evaluating Knowledge, Perceptions, and Clinical Practices (KPCP) regarding HPV and HPV-OPC, originally developed for English-speaking US dental and dental hygiene student populations, and evaluate the applicability and reliability of a Latin American Spanish translation [24]. The primary aim of the study was to perform pretesting of the adapted instrument and assess the reliability of the survey subscales using Cronbach alpha. The secondary aim of the study was to examine the translated survey for linguistic and cultural equivalency using qualitative open-ended questions and in-person discussions.

## Methods

The present study was conducted by researchers from the University of Utah School of Dentistry in Salt Lake City, Utah in the US and the Autonomous University of Baja California’s Faculty of Dentistry in Tijuana, Mexico. Huntsman Cancer Institute and University of Utah School of Medicine also supported this research.

### Questionnaire development

A previously developed questionnaire for English-speaking dental students was utilized for cross-cultural adaptation for Spanish-speaking dental students in Latin America. Using Beaton et al. 2000 as a guideline for cross cultural adaption, and described in Additional file 1: Appendix 1, the process occurred in five general stages [28]. In stage 1 (initial forward translation), forward translation of the previously validated HPV questionnaire was done by two native Spanish-speaking members of the research team certified as bilingual translators (LMPs) who are also fluent in the source language. In stage 2 (synthesis and reconciliation), the initial version was evaluated for semantic equivalence by the appointed in-country investigator (AV), who also served as an editor and highlighted grammatical and typographic errors. Stage 3 was not included, as no back translation was performed. Stage 4 included an expert committee review and reconciliation of forward translation concerns via a discussion between appointed members of the research team, and a final version of the translated survey was made when all concerns were resolved. In stage 5 (pretesting), a pretest of the resulting translated version, of the multi-scale survey, was performed among first-year dental students in two Latin American Countries.

The HPV-OPC Knowledge, Perceptions, and Clinical Practices (KPCP) Spanish Version (SV) multi-scale survey consisted of 147 items, of which 106 items were included in the final analysis. Items made up seven subscales: (1) HPV knowledge, (2) HPV-OPC knowledge, (3) HPV vaccine knowledge, (4) Barriers, (5) Clinical Procedures, (6) Scope of Practice, and (7) Curriculum Evaluation. The questionnaire also included demographic questions. The questions had a variety of response types, including true/false/don’t-know, multiple choice, check all that apply, yes/no, and Likert scale. While 30–40 respondents are often considered a sufficient sample size for cross-cultural adaptation studies [28–30], we estimated sample size of 18–22 respondents was needed, for a power of 0.9 and 14–17 for a power of 0.8, respectively using the Bonett formula [31, 32] (performed in Microsoft Excel, 2019) assuming a Cronbach alpha coefficient equal to zero, for a minimum effect size of 0.70, with a 95% probability of type I error (α = 0.05).

### Questionnaire pretesting

Approval from University of Utah’s and Institutional Review Board (IRB) was obtained prior to the present study. Throughout Latin America, email invitations were sent to eight universities to participate in this pretest of the cross-culturally adapted HPV questionnaire, from which two agreed to participate; one program was located in Colombia (Facultad de Odontología de la Universidad de Antioquia) and the other, in Mexico (Facultad de Odontología de la Universidad Autónoma de Baja California, Tijuana campus). The participating two schools are located in geographical regions where dental student’s HPV-OPC knowledge has not been evaluated before. Eligible participants were first year dental (D1) students as they perform oral health educational duties as part of their program requirements. Convenience sampling and study procedures were conducted by two of the study investigators (Colombia-LMP; Mexico-AV) according to the IRB-approved study protocol in both sites to ensure consistency. First, study investigators gave a short oral presentation about the research project to D1 students. Students were then instructed on how to properly answer the questions and then completed the questionnaire at their own pace. The questionnaire was administered in pencil-and-paper format, and then study investigators manually entered the data into Vanderbilt University’s Research Electronic Data Capture (REDCap) program for data management. Participation was voluntary and completion of the questionnaire served as consent.

Students took between 30 to 40 min to complete the questionnaire, after which, they participated in an in-person discussion for about 30 min. During the in-person discussion, issues related to the survey questions were reviewed to gain additional feedback. Participants also provided their overall impressions of the instrument, suggested changes, shared what they liked or disliked about the instrument, and provided feedback on the order of questions. D1 students were incentivized to participate in the study by holding three raffles per University with the opportunity to win clinical dental instruments. Raffles were done immediately after participants finished the questionnaire and discussion.

Cronbach’s alpha was used to measure the internal consistency of the HPV-OPC-KPCP-SV instrument. The higher the Cronbach’s alpha, the more related the items of the instrument are to each other; a low Cronbach’s alpha may indicate that the items of the instrument are unrelated, a low number of questions are assessed, or that heterogeneous constructs are included. A Cronbach’s alpha threshold of 0.7 and higher was employed as suggested by George and Mallery (2003) [33]. Analyses were conducted using SAS software, Version 9.4, RStudio v.1.4.1717 (cocron library), and Microsoft Excel 2019.

## Results

A total of *N* = 114 Latin American dental students participated in the present study: n = 86 attending Facultad de Odontología de la Universidad Autónoma de Baja California, Tijuana and n = 36 attending Facultad de Odontología de la Universidad de Antioquia in Colombia. The majority of participants were female (61%), Hispanic/Latino(a)/Spanish (91%), and had at least an undergraduate associate and/or bachelor’s degree prior to entering their dental program (100%). Table 1 contains a complete breakdown of participant demographics.

**Table 1:** Demographic characteristics of participants^1^; N = 122 [N(%)]

Sex	
Female	70 (61.4%)
Male	44 (38.6%)
Age^2^	
18–29	114 (100%)
Religion	
Agnostic	4 (3.5%)
Atheist	3 (2.6%)
Buddhist	2 (1.8%)
Catholic	67 (58.8%)
Christian	4 (3.5%)
Jehovah’s witness	2 (1.8%)
Jewish	1 (0.9%)
Mormon/LDS	3 (2.6%)
Protestant	3 (2.6%)
Other	4 (3.5%)
Nothing in particular	6 (5.3%)
Prefer not to answer	11 (9.6%)
Did not answer	4 (3.5%)
Prior degrees earned^3^	
Associate degree	24 (21.1%)
Bachelor’s degree	90 (78.9%)
Master’s degree	2 (1.8%)
Doctorate degree	1 (0.9%)

^1^Among dental students attending Facultad de Odontología de la Universidad Autónoma de Baja California, Tijuana (n = 86) or Facultad de Odontología de la Universidad de Antioquia in Colombia (n = 36)

^2^Participants < 18 years old, or did not provide an age, were excluded from analysis (n = 8, 7% of all who completed a survey)

^3^Participants were able to select all prior degrees earned before entering a dental program and percentages sum to greater than 100%

Table 2 contains a summary of the HPV-OPC-KPCP-SV scale reliability and correlation coefficients, the type of analysis performed, and the number of questions included in each scale. The HPV, HPV-OPC, and HPV vaccine knowledge subscales demonstrated acceptable to good internal consistency, the Cronbach’s alpha (α) was 0.83, 0.75, and 0.86 respectively. The Barriers subscale had a Cronbach’s alpha of 0.93. The Clinical Procedures subscale focused on factors surrounding dental students’ hypothetical clinical practice procedures and had a Cronbach’s alpha of 0.87. The Scope of Practice scale had a Cronbach’s alpha of 0.93. The Curriculum Evaluation scale had a Cronbach’s alpha of 0.23. The questions used for these analyses are shown in Additional file 1: Appendix 2.

**Table 2:** Summary of reliability and correlation coefficients of subscales

			Cronbach alpha^1^	95% CI	
Subscales^2^	Items	N^2^		Lower bound	Upper bound
1. HPV knowledge	22	93	0.83	0.78	0.88
2. HPV-OPC knowledge	13	103	0.75	0.67	0.82
3. HPV vaccine knowledge	25	85	0.86	0.81	0.90
4. Barriers	24	96	0.93	0.90	0.95
5. Clinical procedures	5	74	0.87	0.81	0.91
6. Scope of practice	4	105	0.93	0.90	0.95
7. Curriculum evaluation	6	58	0.23	− 0.12	0.50

^1^Raw Cronbach Alpha reported

^2^The same coding was utilized for calculation as presented and collected (as numeric codes) in the questionnaire. The differences number of respondents (N) utilized for alpha calculations due to missingness of responses in subscale items, such that Cronbach's alpha calculated across respondents with complete data for the subscale items under review

Qualitative comments from students (data not shown) were also concurrently reviewed. All of the students who participated in this study were in their first year of Dental School and it was a common statement that they had not yet taken the oral pathology class, were they would learn information related oral diseases. It was suggested that instead of using the word **“**verdad**”** in true or false questions, to change it to **“**verdadero**”**, as the first word means truth and the latter means true. In Spanish speaking countries, “verdadero**”** is used for these types of questions. Also highlighted by several participants, important grammatical errors, such as missing accents, which can change the context of a question and can cause the participant to re-read every question, including missing backwards interrogation question marks (¿) applied to the beginning of questions.

The average number of correctly answered HPV knowledge questions (44.76%, SD 15.40) and HPV vaccination knowledge questions (41.39%, SD 18.18) among all participants was low overall (44.45%, SD 15.07). As shown in Additional file 1: Appendix 3, the three HPV knowledge questions with the lowest percent of participants answering correctly (all under 9% answered correctly) included lack of knowledge about most HPV infections spontaneously resolving (7.1%), HPV types that cause cervical cancer versus genital warts (8.40%), and the age group for which HPV incidence is highest (8.55%). As shown in Additional file 1: Appendix 4, the three HPV vaccination knowledge questions with the lowest percent of participants answering correctly (all under 20% answered correctly) included lack of knowledge about the optimal age group for vaccination among males (10.7%), the effectiveness of the vaccine over time (15.0%), and receipt of the vaccine after an abnormal Pap smear/Pap test (18.0%) (Additional file 2, Additional file 3, Additional file 4, Additional file 5).

## Discussion

The present study performed the initial steps of a cross-cultural adaptation of a previously validated English version HPV survey, and aimed to evaluate internal consistency of the HPV-OPC Knowledge, Perceptions, and Clinical Practices (KPCP) subscales of the Spanish Version (SV) in a sample of Latin American dental students. The two schools that participated in the SV pretest were both good candidates to test the HPV-OPC-KPCP-SV instrument and measure internal consistency, since their HPV-OPC knowledge had not been evaluated before. The main findings indicate high internal consistency of most subscales in the instrument. Culturally, the survey also demonstrated strong practical relevance for students in Latin American countries, as participants expressed interest in acquiring knowledge related to the topic through open-ended questions. Feedback received from students suggest some minor orthographical and grammatical corrections.

In general, there was evidence of equivalence between the HPV-OPC-KPCP-SV and the English version.

Compared to the alpha coefficients of the English version in our previous work [24], HPV-OPC-KPCP-SV had a higher internal consistency in the following subscales: HPV Knowledge, HPV Vaccine Knowledge, and Scope of Practice. The English version had Cronbach’s alpha coefficient of 0.71 and 0.79 for HPV Knowledge, and HPV Vaccine Knowledge, respectively. These two categories in the English version had lower value of Cronbach’s alpha coefficient, which may due to differences in the sample population and not a characteristic of the instrument. The English version had two items for Scope of Practice and was analyzed using Spearman correlation, which resulted in an alpha coefficient of 0.71, while the SV was not directly comparable because the same questions were analyzed as part of the 24-item Barriers subscale using Cronbach’s alpha (0.93). The SV Scope of Practice four-item subscale (Cronbach’s alpha = 0.93) most similarly resembled the English version Willingness to discuss, recommend, or administer the HPV vaccines two-item subscale (Spearman Correlation Alpha = 0.85) and was not directly comparable due to item and calculation differences. There was no English version equivalent of the SV six-item Curriculum Evaluation subscale and the low internal consistency (Cronbach’s alpha = 0.23) may be due to differences in population characteristics, or differences in curriculum between dental schools in the two Latin American countries. To improve the internal consistency for an HPV Curriculum Evaluation subscale, senior dental students, with program curriculum experience, should be surveyed and analyzed separately by school and/or country. This analysis would assist researchers in understanding which questions in Curriculum Evaluation must be revised and adapted to the dental schools where the survey will be administered.

Previous researches in Latin America have evaluated different aspects related to HPV-OPC in dental students [34]. Each research group developed a questionnaire regarding dental students’ demographic, sexual habits, sexual-related pre-existing pathologies, and HPV-OPC knowledge. Two studies claimed that their questionnaires were validated [34, 35]. Although, there are no statistical reports and references in these studies that allows us to evaluate and compare the validation process of their questionnaires with ours. The description provided by the researchers were as followed: Medina et al. reported that experts validated their questionnaire with high reliability; on the other hand, Lama-González’s group simply reported the administration of an HPV knowledge assessment and questionnaire among dental students of La Facultad de Odontología de la Universidad Autónoma de Yucatan. In both studies, no reports were found in the publications about Cronbach’s alpha coefficient or any statistical analysis used to validate their questionnaires [34, 35].

Even though the previous studies listed above were valuable in assessing dental students’ knowledge regarding HPV-OPC, they did not demonstrate any methodological steps needed to claim validity of their new scales. HPV-OPC-KPCP-SV is the first to describe their validation methodology. Besides assessing dental students’ knowledge, perception, and clinical practice, the current survey also assessed the HPV-OPC curriculum in dental schools. The study results reveal a great opportunity for curriculum evaluation and development among Latin American dental schools and can be used to further implement research in Latin American countries regarding HPV-OPC. By comprehensively evaluating dental students’ knowledge, perception, curriculum, and clinical practice, the barriers that prevent or limit the discussion of HPV-OPC and HPV vaccination with patients can be identified and mitigated. Knowing these barriers provides the foundation to propose a guide and/or intervention that would aim to increase HPV-OPC education among patients and increase HPV vaccination rates in Latin America.

### Limitations

The HPV-OPC-KPCP-SV survey appears to have been successfully translated from English to Spanish. Moreover, the reliability and cross-cultural applicability is appropriate to be implemented in dental students from Latin America countries. However, in interpreting the results, it is important to take the study’s limitations into account. Despite the success of the pretesting of the HPV-OPC-KPCP-SV, the study was conducted in only two countries: Mexico and Colombia, thus findings may not generalize to other Latin American countries. Nevertheless, people from different cultural and demographic background were included in the present study and reliability was high suggesting that generalizability may be appropriate. Additional research is needed to support this claim.

The present study administered one survey in a cross-sectional design meaning Cronbach’s alpha was the only feasible reliability measure that could be assessed. Due to the research team’s limited access to the dental students, funding, and logistical constraints, the students were only asked to complete the survey once and the present study could not measure test–retest reliability. Concurrent validity or discriminant reliability measures were not possible as the students were only asked to complete one survey. Floor and ceiling effects are also possible limitations in surveys of this type. Administering this survey to a wider range of other dental students and professionals, both in experience and knowledge, and at multiple time points would reduce these limitations.

Religion and religiosity have been found to be important factors of HPV vaccination receipt, discussions, attitudes, and knowledge. HPV vaccination is often a health decision that is made on behalf of the patient by parents and/or caregivers, not considered required, and is ideally administered starting at age 9–11 years old, prior to sexual debut. HPV is often viewed as a sexually transmitted disease and that HPV vaccination is not needed for individuals who are in, or will only be in, monogamous relationships. Cronbach’s alpha can be considered as a characteristic of the sample and it is plausible that dimensionality of scales may vary by religion and/or religiosity and reported Cronbach’s alphas are under-estimated. Due to sample size, we were unable to effectively determine dimensionality of scales (via factor analysis), although a cursory examination of the HPV knowledge subscale did indicate the scale may not be unidimensional (13 dimensions indicated by the Guttman-Kaiser rule, Eigenvalues > 0) [36] and that the Cronbach’s alpha (0.83) is likely under-estimated (data not shown). A much larger sample size (3 to 20 times the number of variables in the subscale) [37] that allowed for factor analysis of the subscale by religiosity and/or different religious groups would provide insight into the scale dimensionality differences.

After the survey was administered, there was an in-person group discussion. This may have limited the ability of participants to confidently express and share their feedback. Also, it is possible that the participants’ responses could have been influenced by social desirability and conformity bias. Since participants were relatively new to the dental school academic environment, they may have been knowingly or unknowingly responding to items while taking into consideration their peers or the researchers’ perceptions of their responses. This was mitigated by an anonymous comment field that allowed the participants, who may feel uncomfortable to discus in the in-person group discussion, opportunities to provide their feedback. However, these biases could still exist.

We observed that participants answered an average (mean) of 44.76% (15.40 SD) of all HPV knowledge subscale questions correctly and 41.39% (18.18 SD) of HPV vaccination knowledge subscale questions correctly. Since the participants were first-year students, we expected knowledge scores to be low and that missingness would not be random as some students might feel uncomfortable answering some questions in this pretest due to the limited patient hours and the knowledge regarding cancer-related topics. Likewise, participants had not completed oral pathology curriculum prior to the pretest, meaning caution should be taken when interpreting the knowledge sections’ accuracy or internal consistency. This limitation of knowledge may also be the reason why the Curriculum Evaluation had a low alpha coefficient.

Future research should focus on the evaluation of knowledge that senior dental students (D4 and D5) as well as dentists and dental hygienists have about HPV Knowledge, HPV-OPC Knowledge, HPV Vaccine Knowledge, Barriers, Clinical Procedures, Scope of Practice, and Curriculum Evaluation in order to identify and overcome real or perceived barriers. This could take the form of offering continuing education courses, workshops, or changes in dental school curriculums. Subsequent studies should focus on further validation of the HPV-OPC-KPCP-SV and include assessment of additional reliably measures.

## Conclusions

Aside from assessing the linguistic and cultural equivalency of the Spanish version of the survey, the present study found high internal consistencies for the majority of subscales examining HPV Knowledge, HPV-OPC Knowledge, HPV Vaccine Knowledge, Barriers, Clinical Procedures, and Scope of Practice.

The process of a Spanish translation of the HPV-OPC-KPCP-SV survey demonstrated initial reliability and applicability for the assessment of the knowledge, perceptions, and clinical practices regarding HPV-OPC for dental students in Latin America. However, this is only a first step, the next step would be to do further reliability and validity assessments using a larger sample. The present study has implications for dental HPV curriculum development and may be used to assess needs for improving the quality of dental practice among our future dental health professionals in the area of HPV education, HPV vaccination, and detection of HPV-OPC cancers at early stages.

## Supplementary Information

**Additional file 1:** Survey items and flow chart document.

**Additional file 2:** Subscales analyzed for reliability.

**Additional file 3:** Complete survey.

**Additional file 4:** Cross-cultural adaptation flow chart.

**Additional file 5:** Anonymous participants responses.

## Data Availability

The datasets used and/or analyzed during the current study are available from the corresponding author on reasonable request. Corresponding author: Deanna Kepka, PhD, MPH deanna.kepka@hci.utah.edu.
