# Supplementary material for: Cross-cultural adaptation of a Spanish version of a previously validated HPV survey that evaluates dental students’ knowledge, perception and clinical practices in Latin America
Source: BMC Oral Health. 2022 Mar 14;22:72. doi: 10.1186/s12903-022-02108-2 (PMC8922929; doi:10.1186/s12903-022-02108-2)
Supplement: Supplementary file 1 — Additional file 1: Survey items and flow chart document. [file 12903_2022_2108_MOESM1_ESM.docx]

Appendix 1. Flow chart of the cross-cultural adaptation process. A previously validated survey for assessing dental students’ knowledge of, perception of, and clinical practices for HPV, HPV vaccination, and oropharyngeal cancer screening was adapted from English for Spanish speakers in Latin America. Certified bilingual translators (LMPs) and an appointed in-country investigator (AV) worked with the research team to adapt the instrument. Figure adapted from Beaton et al. (2000)

| Appendix 2. Subscale items analyzed for reliability | | | |
| --- | --- | --- | --- |
| **1. HPV knowledge** | | | |
| **Item (Spanish)** | **Item (English)** | **Item-to-Total Scale Correlation** | **Cronbach Alpha if Item Deleted** |
| Hay muchos tipos de Virus de Papiloma Humano (VPH) | There are many types of Human Papillomavirus (HPV) | 0.35 | 0.82 |
| VPH es una infección bacteriana | HPV is a bacterial infection | 0.36 | 0.82 |
| Una persona puede estar infectado con VPH sin saberlo | A person can have HPV without knowing it | 0.28 | 0.83 |
| Generalmente, casi todas las infecciones de VPH se resuelven espontáneamente después de dos años | Generally, most HPV infections resolve spontaneously within 2 years | 0.37 | 0.82 |
| El VPH puede ser transmitido por contacto sexual | HPV can be transmitted via sexual contact | 0.33 | 0.82 |
| Una persona sin verrugas genitales puede transmitir el VPH | A person can transmit HPV even if a genital wart is not present | 0.55 | 0.81 |
| VPH puede causar cáncer orofaríngeo | HPV can cause oropharyngeal cancer | 0.45 | 0.82 |
| VPH puede causar herpes | HPV can cause herpes | 0.47 | 0.82 |
| VPH puede causar VIH/SIDA | HPV can cause HIV/AIDS | 0.52 | 0.81 |
| VPH puede causar verrugas genitales | HPV can cause genital warts | 0.54 | 0.81 |
| VPH puede causar cáncer de cérvix | Some types of HPV cause cervical cancer | 0.53 | 0.81 |
| La tasa de VPH es más alta en mujeres de 30 años | The rate of HPV is highest among women in their 30s | 0.23 | 0.83 |
| La displasia relacionada con VPH es mas común en personas fumadoras | HPV related dysplasia occurs more commonly in smokers | 0.25 | 0.83 |
| VPH puede causar resultados anormales del examen Papanicolaou | HPV can cause an abnormal Pap smear test/Pap test in women | 0.20 | 0.83 |
| Las verrugas genitales son causadas por el mismo tipo de VPH que causa cáncer de orofaríngeo | Genital warts are caused by the same HPV type(s) that cause oropharyngeal cancer | 0.47 | 0.82 |
| Las verrugas genitales son causadas por el mismo tipo de VPH que causa cáncer de cérvix | Genital warts are caused by the same HPV type(s) that cause cervical cancer | 0.46 | 0.82 |
| Casi todos los canceres cervicales son causados por el VPH | Almost all cervical cancers are caused by HPV | 0.46 | 0.82 |
| Usar condón disminuye la posibilidad de contraer y de transmitir el VPH | Using a condom will decrease the chance of transmitting/acquiring HPV | 0.33 | 0.82 |
| Inclusive después de ser vacunado contra el VPH el uso de condón puede proveer protección de otras enfermedades sexualmente transmisibles y/o infecciones de VPH | Even after HPV vaccination, condoms continue to provide protection against future sexually transmitted infections (STIs) and/or HPV infections | 0.24 | 0.83 |
| Los antibióticos puedes curar el VPH | Antibiotics can cure HPV | 0.43 | 0.82 |
| El cáncer orofaríngeo como resultado del consumo de tabaco (cigarrillos) es una causa de muerte más común que el cáncer orofaríngeo causado por el VPH | Oropharyngeal cancer caused by smoking is more deadly than oropharyngeal cancer caused by HPV | 0.34 | 0.82 |
| Las etapas tempranas de cáncer orofaríngeo asociado con VPH son a menudo asintomáticas | Early stages of HPV oropharyngeal cancer are often asymptomatic | 0.31 | 0.82 |
| **2. HPV-OPC knowledge** | | | |
| **Item (Spanish)** | **Item (English)** | **Item-to-Total Scale Correlation** | **Cronbach Alpha if Item Deleted** |
| En un consultorio odontológico, ¿qué tan seguido debe un paciente recibir un examen oral y de cabeza y cuello para evaluar lesiones que pueden ser cancerosas? | In the dental office, how often should a patient receive an oral, head, and neck cancer examination? | 0.36 | 0.73 |
| ¿En qué grupo de edades se debe realizar examinación oral y de cabeza y cuello para evaluar lesiones que pueden ser cancerosas? | Which age group(s) should an oral, head, and neck cancer examination be performed on? | -0.45 | 0.75 |
| ¿Qué grupo étnico/racial tiene la tasa más alta de cáncer orofaríngeo? | Which ethnic/racial background has the highest rate of oropharyngeal cancer? | 0.40 | 0.73 |
| ¿En cuál grupo de edades se tiene la tasa más alta de cáncer orofaríngeo? | Which age group has the highest rate of oropharyngeal cancer? | 0.57 | 0.70 |
| ¿Cuál sexo/genero tiene la tasa más alta de cáncer orofaríngeo? | Which sex/gender has the highest rate of oropharyngeal cancer? | 0.36 | 0.73 |
| ¿Cuál grupo de edades se tiene la tasa más alta infección con VPH? | Which age group has the highest rate of HPV infections? | 0.44 | 0.72 |
| ¿Qué porcentaje de cáncer orofaríngeo es atribuido al VPH? | What percentage of oropharyngeal cancer is attributed to HPV? | 0.45 | 0.72 |
| Durante los últimos 10 años, ¿cuál de todas las áreas de la cavidad oral mencionadas abajo ha presentado la mayor tasa de cáncer orofaríngeo asociado al VPH? | Over the last ten years, which of the following oral locations had the most increase in rate for HPV related oropharyngeal cancer? | 0.37 | 0.73 |
| Durante los últimos 10 años, ¿cuál grupo de edad ha mostrado el mayor incremento de tasa de cáncer orofaríngeo? | Over the last ten years, which age group has shown the most increase in rate for HPV related oropharyngeal cancer? | 0.60 | 0.70 |
| ¿Cuál de estos profesionales son fuentes de conocimiento clínico confiable sobre cáncer orofaríngeo asociado al VPH? | Which of the following professionals are the most reliable clinical sources of HPV and oropharyngeal cancer information? | 0.09 | 0.75 |
| Discutir la asociación entre cáncer orofaríngeo y el VPH es parte del rol del odontólogo | Discussing the link between HPV and oropharyngeal cancer falls within the scope and role of a dental professional. | 0.27 | 0.74 |
| Recomendar a un paciente la vacuna del VPH es parte del rol profesional del odontólogo | Recommending HPV vaccination falls within the scope and role of a dental professional. | 0.33 | 0.74 |
| La administración de la vacuna del VPH en el consultorio odontológico es parte del rol profesional del odontólogo | Administering the HPV vaccines inside the dental office falls within the scope and role of a dental professional. | 0.32 | 0.74 |
| **3. HPV vaccine knowledge** | | | |
| **Item (Spanish)** | **Item (English)** | **Item-to-Total Scale Correlation** | **Cronbach Alpha if Item Deleted** |
| ¿Sabías algo sobre el VPH antes de responder este cuestionario? | Were you aware of the HPV vaccines before taking this survey? | -0.12 | 0.85 |
| Hay unas vacunas que proveen inmunidad en contra ciertos tipos de VPH | There are vaccines that provide immunity against certain types of HPV | 0.41 | 0.85 |
| La efectividad de la vacuna del VPH no disminuye con el tiempo | The effectiveness of the HPV vaccine does not decrease over time | 0.56 | 0.84 |
| Las vacunas del VPH protegen a las mujeres de contraer cáncer cervical asociado al VPH | HPV vaccines can protect women against HPV related cervical cancer | 0.45 | 0.85 |
| Las vacunas del VPH protegen a las mujeres y hombre de contraer cáncer orofaríngeo asociado al VPH | HPV vaccines can protect men and women against HPV related oropharyngeal cancer | 0.47 | 0.85 |
| Las vacunas del VPH protegen a las mujeres y hombre de contraer cáncer anal asociado al VPH | HPV vaccines can protect men and women against HPV related anal cancer | 0.46 | 0.85 |
| Las vacunas del VPH no protegen a un individuo de todos los tipos de VPH | HPV vaccines do not protect an individual from all types of HPV | 0.42 | 0.85 |
| Individuos vacunados en contra del VPH no tienen que preocuparse de tener sexo seguro | Individuals who receive the HPV vaccines do not have to be concerned with practicing safe sex (e.g. using condoms) | 0.41 | 0.85 |
| La vacunación en contra del VPH incrementa la posibilidad de que las personas incurran en actividades sexuales de riesgo | HPV vaccination increases the likelihood of people engaging in risky sexual behaviors (e.g. multiple partners, unprotected sex, etc.) | 0.45 | 0.85 |
| Generalmente, las vacunas del VPH son seguras | Generally, HPV vaccines are safe | 0.46 | 0.85 |
| En general, las vacunas del VPH no causan efectos secundarios serios | In general, HPV vaccines do not cause serious side effects | 0.31 | 0.85 |
| Las vacunas de VPH son muy costosas para personas sin seguro médico | HPV vaccines are expensive for uninsured individuals | 0.39 | 0.85 |
| Las vacunas de VPH son cubiertas por casi todos los seguros médicos | HPV vaccines are covered by most insurance providers | 0.51 | 0.84 |
| Las vacunas de VPH son administradas en una sola dosis | HPV vaccines are administered in one dose | 0.38 | 0.85 |
| Las vacunas de VPH pueden proteger a las mujeres y hombre de las verrugas genitales asociadas a VPH | HPV vaccines can protect men and women against HPV related genital warts | 0.58 | 0.84 |
| Personas con verrugas genitales no pueden ser vacunados con la vacuna del VPH | People who already had genital warts cannot get the HPV vaccines | 0.27 | 0.85 |
| La vacuna del VPH es efectiva solo en personas que no han tenido ningún tipo de contacto sexual | HPV vaccines are only effective for individuals who have never had sex | 0.47 | 0.85 |
| Estar en una relación monógama elimina el riesgo de infectarse con VPH | Being in a monogamous relationship eliminates your risk of HPV infection | 0.21 | 0.85 |
| Mujeres con resultados anormales de Papanicolaou no deben ser vacunas la vacuna del VPH | Women who have had an abnormal Pap smear/Pap test should not receive the HPV vaccines | 0.37 | 0.85 |
| El centro de control y prevención de enfermedades (CDC) recomienda la vacunación de VPH para mujeres y hombres | The Centers for Disease Control and Prevention (CDC) recommends that the HPV vaccines should be administered to both males and female | 0.47 | 0.85 |
| Discutir con sus pacientes sobre VPH le permite tener una conversación con sus pacientes sobre el comportamiento sexual de ellos | Discussing the HPV vaccines provide an opportunity to have a conversation with your patients about their sexual behaviors (e.g. sexual history, practicing safe sex, etc.) | 0.40 | 0.85 |
| Las vacunas de VPH son altamente efectivas para la prevención de precursores de cáncer cervical | HPV vaccines are highly effective at preventing cervical cancer precursors | 0.44 | 0.85 |
| ¿Cuándo es recomendada idealmente la vacuna del VPH? | When is HPV vaccination ideally recommended? | 0.38 | 0.85 |
| ¿Cuál es la edad optima para administrar la vacuna del VPH a las mujeres? | What is the optimal age for HPV vaccination in females? | 0.34 | 0.85 |
| ¿Cuál es la edad optima para administrar la vacuna del VPH a los hombres? | What is the optimal age for HPV vaccination in males? | 0.23 | 0.85 |
| **4. Barriers** | | | |
| **Item (Spanish)** | **Item (English)** | **Item-to-Total Scale Correlation** | **Cronbach Alpha if Item Deleted** |
| Basado en su experiencia como profesional de la salud oral, ¿qué tan difícil o fácil es discutir los siguientes temas con sus pacientes? | Based on your experience, how difficult or easy is it to discuss the following topics with your patients? | -- | -- |
| Abuso (ejemplo: violencia domestica, abuso a menores, abuso adulto mayor) | Abuse (e.g., domestic violence, child abuse, elder abuse) | 0.58 | 0.92 |
| Desordenes alimenticios | Eating disorders | 0.58 | 0.92 |
| Historia de enfermedades sexualmente transmisibles (ETS) | Sexually transmitted infection (STI or STD) history | 0.60 | 0.92 |
| Comportamientos sexuales (ejemplo: sexo oral, sexo anal) | Sexual behaviors (e.g. oral sex, anal sex) | 0.57 | 0.92 |
| Consumo y dependencia de sustancias psicoactivas (tabaco, alcohol, drogas) | Substance use disorders (e.g. tobacco use, alcohol use, illicit drugs) | 0.59 | 0.92 |
| Historial de vacunas | Vaccination history | 0.58 | 0.92 |
| El paciente es del sexo opuesto | The patient is of the opposite sex | 0.54 | 0.92 |
| No hay suficiente tiempo para discutir el tema durante la cita | There is not enough time to discuss this during appointments | 0.64 | 0.92 |
| No me siento cómodo(a) con el tema | I do not feel comfortable with the topic | 0.65 | 0.92 |
| Miembros adicionales del equipo odontológico deberían estar en el consultorio | Additional dental team members would also have to be in the room | 0.57 | 0.92 |
| Problemas de confidencialidad | Confidentiality issues | 0.59 | 0.92 |
| El paciente era un menor de edad | The patient was a minor | 0.61 | 0.92 |
| Hay mucha diferencia de edad (mayor/menor) entre el paciente y yo | There was a large age difference (older/younger) between the patient and me | 0.60 | 0.92 |
| No creo que sea mi papel como profesional de la salud oral discutir la historia sexual de los pacientes | I do not believe it is my role as an oral health professional to discuss a patient’s sexual history | 0.63 | 0.92 |
| El diseño físico de mi consultorio no proporciona suficiente privacidad | The physical layout of my office does not afford enough privacy (e.g., other patients’ presence in a larger exam area, no doors on the exam room, etc.) | 0.60 | 0.92 |
| No tengo suficiente información acerca de las vacunas contra el VPH | I do not have enough information about the HPV vaccine | 0.60 | 0.92 |
| Estoy preocupado por la seguridad de las vacunas contra el VPH | I am concerned with the safety of the HPV vaccines | 0.55 | 0.92 |
| Razones de responsabilidad legal | Liability reasons | 0.51 | 0.92 |
| No creo que sea mi papel como profesional de la salud oral recomendar vacunas contra el VPH a mis pacientes | I do not believe it is my role as an oral health professional to recommend the HPV vaccines to my patients | 0.64 | 0.92 |
| No hay políticas establecidas ni guías profesionales pertinentes en la recomendación de la vacuna contra VPH | There are no established professional policies/guidelines pertaining to recommendation of the HPV vaccines | 0.53 | 0.92 |
| No hay suficiente tiempo para discutir el tema en las citas | There is not enough time to discuss this during appointments | 0.62 | 0.92 |
| No me siento cómodo hablando de la historia sexual con mis pacientes o de temas relacionados | I am not comfortable discussing sexual history/topics with patients | 0.65 | 0.92 |
| Las normas sociales y culturales juegan un papel en la discusión del VPH y de las vacunas contra el VPH en el consultorio odontológico | Social and cultural norms play a role in discussing HPV and the HPV vaccines in the dental office | 0.42 | 0.92 |
| La ideología religiosa del paciente juega un papel en la discusión del VPH y de las vacunas contra el VPH en el consultorio odontológico | A patient's religious ideology plays a role in discussing HPV and the HPV vaccines in the dental office | 0.44 | 0.92 |
| **5. Clinical Procedures** | | | |
| **Item (Spanish)** | **Item (English)** | **Item-to-Total Scale Correlation** | **Cronbach Alpha if Item Deleted** |
| ¿Cuál de las siguientes opciones describe mas apropiadamente su procedimiento normal para conversar con sus pacientes sobre el propósito y resultados de las evaluaciones de cáncer oral y de cabeza y cuello? | Which of the following describes your typical approach in communicating with patients about the purpose and the results of their oral, head, and neck cancer examination? | 0.76 | 0.82 |
| Si usted tuviera que discutir la relación entre VPH y el cáncer orofaríngeo con sus pacientes, ¿cuál de los siguientes puntos cree usted que facilitarían esta conversación? | If you were to discuss the relationship between HPV and oropharyngeal cancer with your patients, which of the following do you think would best facilitate this conversation? | 0.13 | 0.93 |
| ¿Cuál frase describe mejor como usted se comunica con pacientes acerca de las vacunas contra el VPH? | Which statement best describes how you communicate with patients about the HPV vaccines? | 0.88 | 0.78 |
| ¿Cuál frase describe mejor como usted comunica a sus pacientes la conexión de la salud oral y sistémica? | Which statement best describes how you communicate with patients about their oral/systemic health? | 0.85 | 0.79 |
| De los métodos listados a continuación, ¿cuál cree usted produciría la información más certera respecto a la historia sexual de los pacientes? | Which statement best describes how you communicate with patients about their sexual history? | 0.79 | 0.81 |
| **6. Scope of Practice** | | | |
| **Item (Spanish)** | **Item (English)** | **Item-to-Total Scale Correlation** | **Cronbach Alpha if Item Deleted** |
| ¿Usted estaría interesado en participar en un entrenamiento para administrar vacunas contra el VPH en su práctica odontológica? | How willing would you be to participate in a training to administer the HPV vaccines in your dental practice? | 0.77 | 0.93 |
| ¿Usted estaría interesado en participar en un entrenamiento para administrar vacunas contra de la influenza/gripe en su práctica odontológica? | How willing would you be to participate in a training to administer the flu vaccines in your dental practice? | 0.87 | 0.89 |
| Si recibiera entrenamiento, ¿usted estaría interesado en administrar vacunas contra el VPH en su practica odontológica? | If trained, how willing would you be to administer the HPV vaccines in your dental office? | 0.89 | 0.88 |
| Si fuera adecuadamente entrenado, ¿qué tan dispuesto estaría de administrar la vacuna contra la gripe en su consultorio odontológico? | If properly trained, how willing would you be to administer the flu vaccine in your dental office? | 0.81 | 0.91 |
| **7. Curriculum Evaluation** | | | |
| **Item (Spanish)** | **Item (English)** | **Item-to-Total Scale Correlation** | **Cronbach Alpha if Item Deleted** |
| ¿Qué tan buena ha sido su educación en odontología para hacer una evaluación de cáncer oral y de cabeza y cuello? | How well has your dental education prepared you to perform an oral, head, and neck cancer examination? | -0.33 | 0.24 |
| ¿Qué tanta confianza tiene en su habilidad para hacer una evaluación de cáncer oral y de cabeza y cuello? | How confident are you in your ability to perform an oral, head, and neck cancer examination? | -0.04 | 0.23 |
| ¿Con qué frecuencia se ha discutido durante su educación odontológica sobre el VPH y la relación entre el VPH y el cáncer orofaríngeo? | How frequently has HPV or HPV related oropharyngeal cancer been discussed during your dental education? | -0.11 | 0.24 |
| ¿Hasta que punto está de acuerdo con la siguiente declaración: “mi plan de estudios odontológicos debería haber tenido más énfasis sobre la asociación del VPH con el cáncer orofaríngeo? | What is your level of agreement with the following statement: "More emphasis should have been placed on HPV related oropharyngeal cancer education during my dental curriculum." | 0.26 | 0.02 |
| ¿Cuál es el área mas común para realizar biopsia de diagnóstico del cáncer orofaríngeo asociado al VPH? | Where is the most common location for a HPV related oropharyngeal cancer biopsy? | 0.15 | 0.15 |
| Existen estándares o pruebas de rutina para el cáncer orofaríngeo específicamente | There are standards or routine screening tests for oropharyngeal cancer, specifically. | 0.10 | 0.19 |

| **Appendix 3. HPV knowledge^1^ among university dental students in Colombia and Mexico** | | | |
| --- | --- | --- | --- |
| Survey Question | N Missing | Frequency Correct | Percent Correct |
| There are many types of Human Papillomavirus | 1 | 80 | (70.8) |
| HPV is a bacterial infection | 2 | 51 | (45.5) |
| A person can have HPV without knowing it | 4 | 96 | (87.3) |
| Generally, most HPV infections resolve spontaneously | 1 | 8 | (7.1) |
| HPV can be transmitted via sexual contact | 0 | 92 | (80.7) |
| A person can transmit HPV even if a genital wart is not present | 3 | 71 | (64.0) |
| HPV can cause oropharyngeal cancer | 0 | 50 | (43.9) |
| HPV can cause herpes | 0 | 18 | (15.8) |
| HPV can cause HIV/AIDS | 0 | 35 | (30.7) |
| HPV can cause genital warts | 0 | 80 | (70.2) |
| Some types of HPV cause cervical cancer | 2 | 54 | (48.2) |
| The rate of HPV is highest among women in their 30s | 2 | 9 | (8.0) |
| HPV related dysplasia occurs more commonly in smokers | 2 | 15 | (13.4) |
| HPV can cause an abnormal Pap smear/Pap test in women | 2 | 58 | (51.8) |
| Genital warts are caused by the same HPV type(s) that cause oropharyngeal cancer | 2 | 13 | (11.6) |
| Genital warts are caused by the same HPV type(s) that cause cervical cancer | 0 | 10 | (8.8) |
| Almost all cervical cancers are caused by HPV | 2 | 23 | (20.5) |
| Using a condom will decrease the chance of transmitting/acquiring HPV | 1 | 98 | (86.7) |
| Even after HPV vaccination, condoms continue to provide protection against future Sexually Transmitted Infections (STIs) and/or HPV infections | 1 | 100 | (87.7) |
| Antibiotics can cure HPV | 2 | 54 | (48.2) |
| Oropharyngeal cancer caused by smoking is more deadly than oropharyngeal cancer caused by HPV | 0 | 48 | (42.1) |
| Early stages of HPV related oropharyngeal cancer are often asymptomatic | 1 | 47 | (41.6) |
| ^1^Frequency and percent of participants answering HPV knowledge survey questions correctly. Participants answered an average (mean) of 44.76% (15.40 SD) of all HPV knowledge subscale questions correctly and 41.39% (18.18 SD) of HPV vaccination knowledge subscale questions correctly. Missing answers were considered, whereby percent correct was calculated using the number of questions answered in the denominator. | | | |

| **Appendix 4. HPV vaccination knowledge^1^ among university dental students in Colombia and Mexico** | | | |
| --- | --- | --- | --- |
| Individual Questions | N Missing | Frequency Correct | Percent Correct |
| Were you aware of the HPV vaccines before taking this survey? | 1 | 54 | (47.8) |
| There are vaccines that provide immunity against certain types of HPV | 1 | 82 | (72.6) |
| The effectiveness of the HPV vaccine does not decrease over time | 1 | 17 | (15.0) |
| HPV vaccines can protect women against HPV related cervical cancer | 0 | 61 | (53.5) |
| HPV vaccines can protect men and women against HPV related oropharyngeal cancer | 1 | 37 | (32.7) |
| HPV vaccines can protect men and women against HPV related anal cancer | 1 | 25 | (22.1) |
| HPV vaccines do not protect an individual from all types of HPV | 1 | 42 | (37.2) |
| Individuals who receive the HPV vaccines do not have to be concerned with practicing safe sex (e.g. using condoms) | 0 | 100 | (87.7) |
| HPV vaccination increases the likelihood of people engaging in risky sexual behaviors (e.g. multiple partners, unprotected sex, etc.) | 1 | 58 | (51.3) |
| Generally, HPV vaccines are safe | 0 | 70 | (61.4) |
| In general, HPV vaccines do not cause serious side effects | 0 | 28 | (24.6) |
| HPV vaccines are expensive for uninsured individuals | 1 | 22 | (19.5) |
| HPV vaccines are covered by most insurance providers | 0 | 49 | (43.0) |
| HPV vaccines are administered in one dose | 0 | 31 | (27.2) |
| HPV vaccines can protect men and women against HPV related genital warts | 1 | 45 | (39.8) |
| People who have already had genital warts cannot get the HPV vaccines | 0 | 32 | (28.1) |
| HPV vaccines are only effective for individuals who have never had sex | 0 | 61 | (53.5) |
| Being in a monogamous relationship eliminates your risk of HPV infection | 0 | 49 | (43.0) |
| Women who have had an abnormal Pap smear/Pap test should not receive the HPV vaccines | 3 | 20 | (18.0) |
| The Centers for Disease Control and Prevention (CDC) recommends that the HPV vaccines should be administered to both males and females | 1 | 54 | (47.8) |
| Discussing the HPV vaccines provide an opportunity to have a conversation with your patients about their sexual behaviors (e.g. sexual history, practicing safe sex, etc.) | 2 | 72 | (64.3) |
| HPV vaccines are highly effective at preventing cervical cancer precursors | 0 | 41 | (36.0) |
| When is HPV vaccination ideally recommended? | 12 | 70 | (68.6) |
| What is the optimal age for HPV vaccination in females? | 4 | 33 | (30.0) |
| What is the optimal age for HPV vaccination in males? | 2 | 12 | (10.7) |
| ^1^Frequency and percent of participants answering HPV knowledge survey questions correctly. Participants answered an average (mean) of 44.76% (15.40 SD) of all HPV knowledge subscale questions correctly and 41.39% (18.18 SD) of HPV vaccination knowledge subscale questions correctly. Missing answers were considered, whereby percent correct was calculated using the number of questions answered in the denominator. | | | |
